# Supplementary material for: Inflammation drives alternative first exon usage to regulate immune genes including a novel iron-regulated isoform of Aim2
Source: eLife. 2021 May 28;10:e69431. doi: 10.7554/eLife.69431 (PMC8260223; doi:10.7554/eLife.69431)

Uncropped WB Images for KO BMDM Cas9 Cell Line

Aim2 Ab

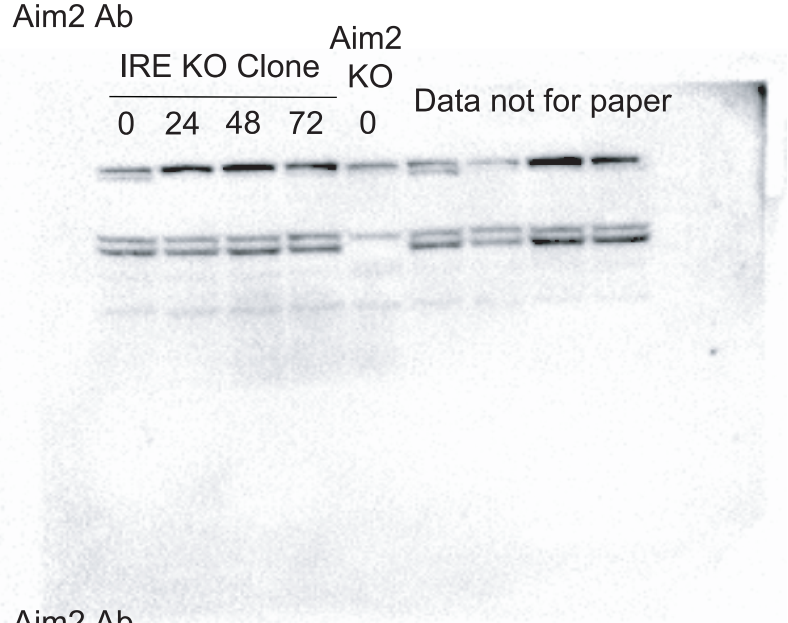

Actin Ab

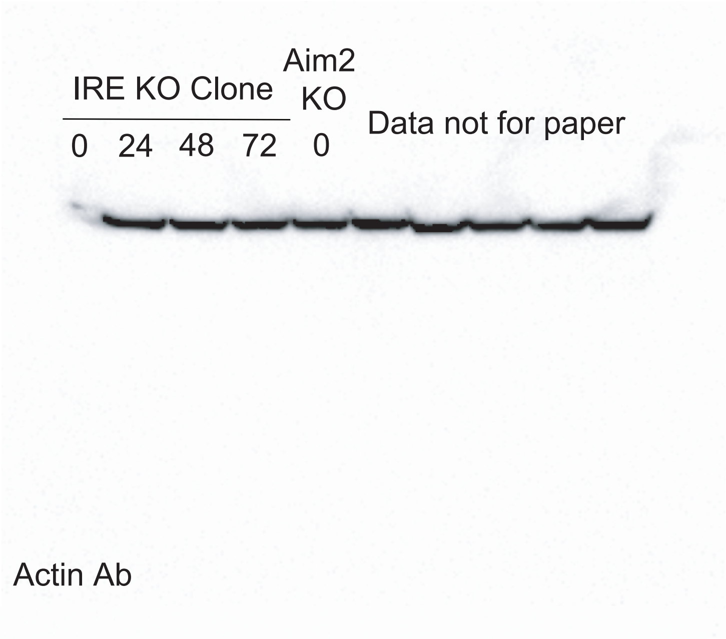

Aim2 Ab

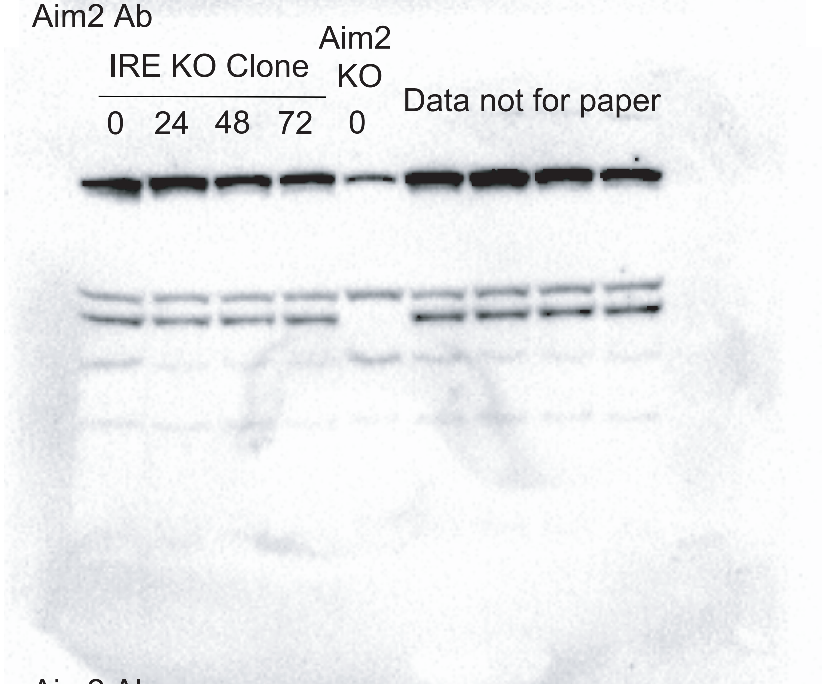

Actin Ab

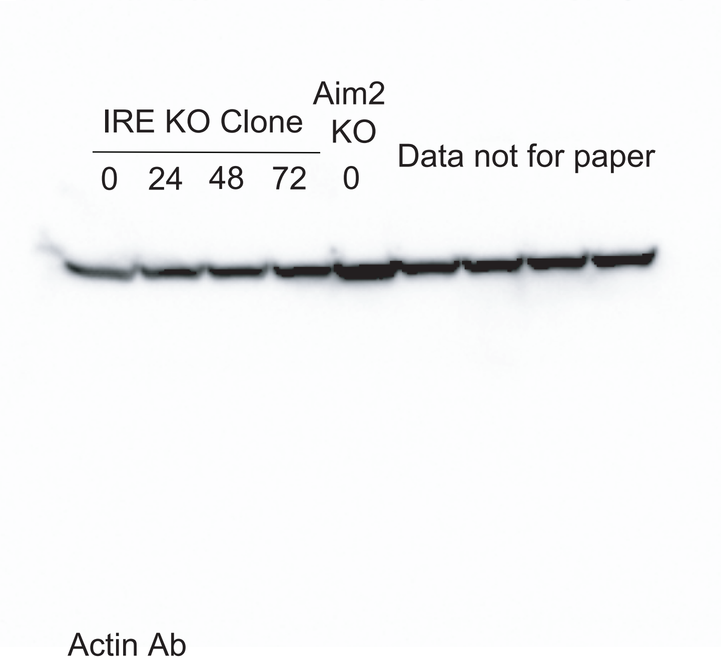

Aim2 Ab

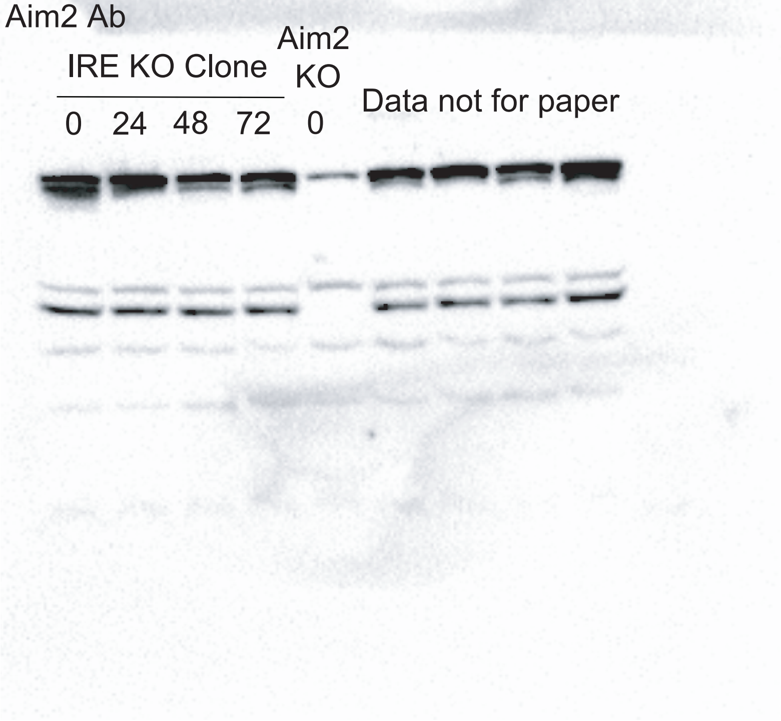

Actin Ab

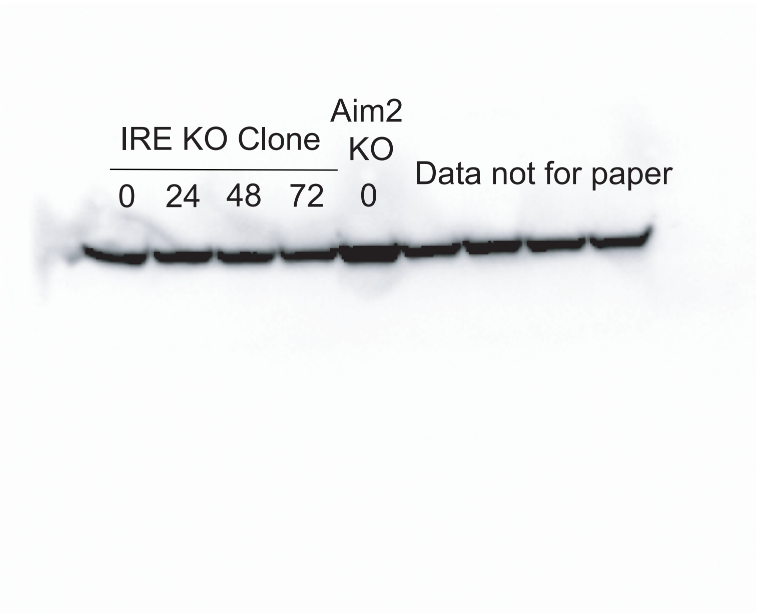

Supplement: Figure 4—figure supplement 4—source data 2. [file elife-69431-fig4-figsupp4-data2.pdf]
